# Is a Perceived Activity-Friendly Environment Associated with More Physical Activity and Fewer Screen-Based Activities in Adolescents?

**DOI:** 10.3390/ijerph14010039

**Published:** 2017-01-03

**Authors:** Jaroslava Kopcakova, Zuzana Dankulincova Veselska, Andrea Madarasova Geckova, Jens Bucksch, Hanna Nalecz, Dagmar Sigmundova, Jitse P. van Dijk, Sijmen A. Reijneveld

**Affiliations:** 1 Department of Health Psychology, Faculty of Medicine, Pavol Josef Safarik University in Kosice, Tr. SNP 1, 040 11 Kosice, Slovak Republic; zuzana.veselska@upjs.sk (Z.D.V.); geckova.andrea.madarasova@upjs.sk (A.M.G.); 2 Graduate School Kosice Institute for Society & Health, Pavol Josef Safarik University in Kosice, Tr. SNP 1, 040 11 Kosice, Slovak Republic; j.p.van.dijk@umcg.nl; 3 Faculty of Physical Culture, Institute of Active Lifestyle, Palacky University Olomouc, Tr. Miru 117, 771 11 Olomouc, Czech Republic; dagmar.sigmundova@upol.cz; 4 Bielefeld University, School of Public Health, PO Box 10 01 31, D-33501 Bielefeld, Germany; jens.bucksch@hbsc.org; 5 The Jozef Pilsudski University of Physical Education, Faculty of Physical Education, Marymoncka 34 Str., 00-968 Warsaw, Poland; hania.nalecz@hbsc.org; 6 Olomouc University Social Health Institute, Palacky University Olomouc, Univerzitni 22, 771 11 Olomouc, Czech Republic; 7 Department of Social Medicine and Public Health, Faculty of Medicine and Dentistry, Palacky University Olomouc, 771 11 Olomouc, Czech Republic; 8 Department of Community & Occupational Medicine, University Medical Center Groningen, University of Groningen, A. Deusinglaan 1, 9713 AV Groningen, The Netherlands; s.a.reijneveld@umcg.nl

**Keywords:** physical activity, screen-based activities, perceived activity-friendly environment, adolescence

## Abstract

*Background:* The aim of this study is to explore if perception of an activity-friendly environment is associated with more physical activity and fewer screen-based activities among adolescents. *Methods:* We collected self-reported data in 2014 via the Health Behavior in School-aged Children cross-sectional study from four European countries (*n* = 13,800, mean age = 14.4, 49.4% boys). We explored the association of perceived environment (e.g., “There are other children nearby home to go out and play with”) with physical activity and screen-based activities using a binary logistic regression model adjusted for age, gender, family affluence and country. *Results:* An environment perceived as activity-friendly was associated with higher odds that adolescents meet recommendations for physical activity (odds ratio (OR) for one standard deviation (SD) change = 1.11, 95% confidence interval (CI) 1.05–1.18) and lower odds for excessive screen-based activities (OR for 1 SD better = 0.93, 95% CI 0.88–0.98). *Conclusions:* Investment into an activity-friendly environment may support the promotion of active life styles in adolescence.

## 1. Introduction

A higher level of physical activity and a lower level of screen-based activities are of major importance for the development of youth and for physical, psychological and socio-emotional health [1,2,3,4]. They offer numerous health benefits to adolescents [1,5]. It is recommended that adolescents participate in physical activity for at least 60 min daily [6] and in screen-based activities for no more than 2 h daily [7]. Recent studies [5,8,9,10] indicated that only a minority of adolescents meet the current guidelines for physical activity and screen-based activities.

There is a growing body of literature that recognizes the importance of various determinants regarding physical activity and screen-based activities, such as, e.g., the level of urbanization [11], and the parents’ own level of physical activity [12]. An environment perceived as activity-friendly may affect both physical activity and screen-based activities, but the mechanism is not clear. Recent evidence from an international cross-sectional study of adults suggests that a physical activity-friendly environment may be important for the promotion of physical activity [3], and this might also affect screen-based activities. However, studies among adolescents within a European context are scarce [13,14].

Taking into account the importance of the how adolescents perceive their environment and the scarcity of evidence, the aim of this study was to explore if perception of an activity-friendly environment is associated with adolescents’ behavior in terms of physical activity and screen-based activities using data from a European context.

## 2. Materials and Methods

### 2.1. Sample and Procedure

The Health Behavior in School-aged Children (HBSC) study is a World Health Organization (WHO) collaborative cross-national study with a standardized methodological approach. All procedures are conducted in accordance with the study protocol prepared by the HBSC International Coordinating Center. Data are collected every four years from a nationally representative sample of 11-, 13- and 15-year-old adolescents from the 45 member countries. The primary sampling units are schools and classes. In some countries specific items are not asked for 11-year-old adolescents. More detailed information about the HBSC methodology can be found in Roberts et al. [15].

We include four countries (Czech Republic, Germany, Poland and Slovakia) that collected data on physical activity and screen-based activities (watching TV, computer use for gaming and non-gaming purposes) and on the optional package about physical activity-related correlates of the social and physical environment (labelled as the ‘perceived environment’). To obtain a comparable sample in all four countries we present data on 13- to 16-year-old school children from the latest survey wave in 2014 and exclude 11-year-old adolescents, as only some countries included these questions for this age category. Each of the country-specific samples was based on a nationally representative randomized cluster (i.e., school level) sampling procedure. In total 13,800 (mean age = 14.4, 49.4% boys) students were recruited. Surveys were administered by the class teachers/trained research assistants during regular class time; participation was voluntary, and confidentiality of the participants was ensured. Response rates varied per country (89.2% in the Czech Republic, 72.5% in Germany, 86.1% in Poland and 78.8% in Slovakia). Non-response was mainly due to illness (Czech Republic, Poland and Slovakia), parental disapproval of the participation of their children (Germany, Poland and Slovakia) and children’s disapproval of the participation in the study (Czech Republic).

The study was conducted in accordance with the Declaration of Helsinki, and protocols were approved in Czech Republic by the Ethics Committee of the Faculty of Physical Culture, Palacky University Olomouc (No. 17/2013), in Germany by the Ethics Committee of the University Hospital Hamburg, in Poland by the Bioethics Committee at the Institute of Mother and Child No. 20/2013 within the project funded by the National Science Center, No. 2013/09/B/HS6/03438, and in Slovakia on 18 June 2012 by the Ethics Committee of the Medical Faculty at the P. J. Safarik University in Kosice (No.: 9/2012) under the project APVV 0032-11.

### 2.2. Measures

We collected data on age and gender by using single questions which were validated in previous HBSC surveys [5,10].

Family affluence was measured using the Family Affluence Scale III (FAS III), which consists of six questions: ‘Does your family own a car, van or truck’ (No/Yes, one/Yes, two or more)? ‘Do you have your own bedroom for yourself’ (No/Yes)? ‘How many computers does your family own’ (None/One/Two/More than two)? ‘How many bathrooms (room with a bath/shower or both) are in your home’ (None/One/Two/More than two)? ‘Does your family have a dishwasher at home’ (No/Yes)? ‘How many times did you and your family travel out of your country for a holiday/vacation last year?’ (Not at all/Once/Twice/More than twice)? We converted the FAS summary scores to a final score, which has a normal distribution and a range from 0 to 1. We then created tertile categories of low (0 to 0.333), medium (0.334 to 0.666) and high (0.667 to 1) socio-economic position [16].

Moderate-to-vigorous physical activity was measured by an item asking adolescents about the number of days over the past week that they were physically active for a total of at least 60 min per day. The question was preceded by an explanatory text that defined moderate-to-vigorous activity as ‘any activity that increases your heart rate and makes you get out of breath some of the time’ and offered examples of such activities (running, inline skating, cycling, dancing, swimming, ice skating, etc.) [5,10]. Responses concerned zero to seven days per week and were classified as sufficient—physical activity of seven days vs. less—insufficient, based on the WHO recommendation [6].

Screen-based activities (during the week), represented by watching TV, playing computer games and working with computer, were computed using three separate items. Watching TV was measured by the question: “How many hours a day, in your free time, do you usually spend watching television, videos (including YouTube or similar services), DVDs, and other entertainment on a screen?” Computer gaming was measured by asking: “How many hours a day, in your free time, do you usually spend playing games on a computer, games console, tablet (like iPad), smartphone or other electronic devices (not including moving or fitness games)?” Computer work was assessed by asking: “How many hours a day, in your free time, do you usually spend using electronic devices such as computers, tablets (like an iPad) or smartphones for other purposes, for example, homework, emailing, tweeting, Facebook, chatting, surfing the Internet” [5,10,17]. In our study we combined these three separate items into one composite variable called screen-based activities. Following the latest evidence-based recommendation of the Canadian sedentary behavior guidelines for children and youth [7], time spent on screen-based activities was dichotomised as excessive at least in one screen-based activity (two and more hours/day) and non-excessive (less than 2 h).

Perceived environment was measured using a five-item scale derived from the European Youth Heart Study [14]. Respondents were asked to respond to the following statements: “It is safe to walk or play alone in my neighborhood during the day.”; “There are other children nearby home to go out and play with.”; “There is somewhere at home I can go out and play.”; “There are playgrounds or parks close to my home where I can play.” and “At school there are playgrounds or fields where I can run around.”. Each item had three response categories, i.e., definitely yes, undecided, definitely no.

### 2.3. Statistical Analyses

First, we used descriptive analyses to show the frequencies of gender, family affluence, country, moderate-to-vigorous physical activity and screen-based activities in the whole sample. Next, we analyzed the factor structure of the perceived environment. Principal Component Analysis (PCA) with Varimax rotation was conducted. Following the identification of the final factor model, one latent standardised and normalised variable was created (labelled as the ‘perceived environment’) and used in the remaining analyses. The higher adolescents scored in each item of the activity-friendly environment, the higher the levels of activity-friendly environment that they reported. Third, we performed logistic regressions on the association of the perceived environment with moderate-to-vigorous physical activity and screen-based activities, adjusted for age, gender, family affluence and country. Analyses were conducted with SPSS v21 (IBM, Armonk, NY, USA).

## 3. Results

The background characteristics of the sample are presented in Table 1.

Next, the PCA yielded one latent standardized and normalized variable (labeled as the ‘perceived environment’) (Kaiser-Meyer-Olkin Measure of Sampling Adequacy value was 0.68; Bartlett’s test of sphericity was statistically significant (*p* < 0.001)). Table 2 presents the perceived environment factors represented by one component (labeled as Perceived environment).

Table 3 presents the odds ratios (OR) and 95% confidence intervals (CI) for the perceived environment, with physical activity and screen-based activity from the binary logistic regression analyses adjusted for age, gender, family affluence and country. A sufficient moderate-to-vigorous physical activity was associated with the adolescent perception that the environment promotes physical activity (OR for one standard deviation (SD) change = 1.11, 95% CI 1.05–1.18). We also found a significant association between excessive screen-based activities and perceived environment (OR for 1 SD better = 0.93, 95% CI 0.88–0.98). The perception of an activity-friendly environment was associated with lower odds of excessive screen-based activities among adolescents.

## 4. Discussion

We found that a higher perception of an activity-friendly environment was significantly associated with higher odds that adolescents meet the recommendations for physical activity among the four European countries studied. Furthermore, we found that a perception of the environment as more activity-friendly was associated with lower odds of excessive screen-based activities among adolescents. Moreover, our study also showed that younger adolescents, boys and adolescents from highly affluent families tend to meet physical activity recommendations and that older adolescents and boys tend to do more excessive screen-based activities in four European countries.

Our study confirmed the findings of previous studies [13,18,19,20] showing that promoting adolescents’ physical activity through a perceived activity-friendly environment is associated with achieving the daily recommended physical activity levels. In contrast, a study by Bringolf-Isler et al. [21] in Swiss children found that environmental factors were not associated with physical activity. As similar associations were found in most other studies, it might be expected that a more general pattern is present. However, the discrepancy with the study by Bringolf-Isler et al. [21] also suggests that other possible factors might play a role that causes these differences and should be explored further.

We found that perception of the environment as activity-friendly was significantly associated with excessive screen-based activities among adolescents. This is in line with previous research, which suggests that the built environment may be key in promoting an active lifestyle among adolescents [22]. Our findings are in line with previous studies among Australian children and youth [23,24]. Veitch et al. [23] showed that parental satisfaction with the quality of their local parks was associated with less computer time, and that greater public open spaces were associated with less TV viewing among children. However, research on the association between adolescents’ perception of an activity-friendly environment and screen-based activities within the European context is scarce and comparison with the previously mentioned Australian studies might be problematic based on different cultural backgrounds.

Moreover, it might be inferred that more screen-time activities lead to less physical activity, but screen-time activities and physical activity are recognized as independent constructs, and various studies have highlighted the relative independence of these two behaviors [25]. In that sense we are using two separate outcome variables that are intertwined. It is important to indicate that moderate-to-vigorous physical activity and screen-based activities among adolescents are not strongly related to each other, so using them as separate entities/outcomes might be justified.

The main strengths of this study are its large sample size and the representative international dataset. However, some limitations have to be discussed as well. A first limitation is the cross-sectional design of our study, which hinders conclusive inferences about causality. Our findings therefore need to be confirmed in longitudinal or experimental studies. Another limitation is that we used subjective self-reports for measuring the level of moderate-to-vigorous physical activity and screen-based activities, which may lead to their misclassification and biased estimates. Next, using a binary outcome of moderate-to-vigorous physical activity might be pointed out as a limitation, but the distribution of the variable does not allow many other alternatives. However, more classifications (i.e., use of cut-offs) exist for being physically active vs. not being physically active. For the purpose of the present study we adhered to a frequently used classification, i.e., the WHO recommendation (seven days = active vs. less = inactive) [6].

We found that a higher perception of an activity-friendly environment was associated with higher odds that adolescents will meet recommendations for physical activity and with lower odds of excessive screen-based activities. These findings may guide actions to increase physical activity and reduce screen-based activities of adolescents, but they need confirmation in longitudinal or experimental research.

Moreover, it seems that adolescents’ moderate-to-vigorous physical activity and screen-based activities are linked; adolescents can be active and sedentary on the same day. Therefore, it is important to take a closer look at investments into an activity-friendly environment to promote moderate-to-vigorous physical activity and to reduce screen-based activities in adolescence and to support their active lifestyle. However, based on existing age and gender differences in the prevalence of physical activity and screen-based activities, it seems that such activities among adolescents should take into account different approaches for the investment into an activity-friendly environment.

## 5. Conclusions

The perceived environment is associated with adolescents’ physical activity and their screen-based behavior. Investment into an activity-friendly environment might, among other factors, play a role in the promotion of an active lifestyle in adolescence.

## Figures and Tables

**Table 1:** Descriptive characteristics for the whole sample.

		N (Valid %)
Gender	boys	6822 (49.4)
	girls	6978 (50.6)
Family Affluence	low	3844 (29.5)
	medium	5517 (42.4)
	high	3659 (28.1)
Country	Slovakia	3142 (22.8)
	Czech Republic	3481 (25.2)
	Germany	4174 (30.2)
	Poland	3003 (21.8)
Physical activity	sufficient (seven days)	2182 (16.9)
	not sufficient	10,739 (83.1)
Screen-based activities	excessive (2+ h/day)	10,967 (83.2)
	non-excessive	2212 (16.8)

Notes: Number of missing cases per variable: Gender—0; family affluence—780; country—0; physical activity—879; screen-based activities—621.

**Table 2:** Outcomes of a principal component analysis with Varimax rotation of the perceived environment factors in the sample.

	Component 1 Perceived Environment
There are other children near home to go out and play with	0.68
There is somewhere at home I can go out and play with	0.69
It is safe to walk or play alone in my neighborhood during the day	0.45
There are playgrounds or parks close to my home where I can play	0.49
At school there are playgrounds or fields where I can run around	0.43

**Table 3:** Associations of the perceived environment with physical activity and screen-based activity, adjusted for age, gender, family affluence (FAS) and country: Odds ratios (OR) and 95% confidence intervals (95% CI) from binary logistic regression.

		Physical Activity	Screen-Based Activities
		OR (95% CI)	OR (95% CI)
Age (per year)		0.86 (0.82–0.91) ***	1.30 (1.24–1.36) ***
Gender	girls	1 (ref.) ***	1 (ref.) **
	boys	1.62 (1.46–1.80)	1.17 (1.06–1.30)
FAS	low	1 (ref.) ***	1 (ref.) ^ns^
	medium	1.12 (0.98–1.29)	1.12 (0.99–1.27)
	high	1.60 (1.39–1.85)	1.07 (0.93–1.22)
Country	Slovakia	1 (ref.) ***	1 (ref.) ***
	Czech Republic	1.48 (1.26–1.72)	0.80 (0.69–0.93)
	Germany	0.86 (0.73–1.01)	0.74 (0.64–0.85)
	Poland	1.49 (1.24–1.79)	0.69 (0.58–0.81)
Perceived environment (1 SD better)		1.11 (1.05–1.18) ***	0.93 (0.88–0.98) **
Negelkerke R square		0.04	0.02

Notes: *** *p* ˂ 0.001; ** *p* ˂ 0.01; ^ns^—not significant; ref. = reference group; SD = standard deviation.
